# Representations of extensions of simple groups

**DOI:** 10.1007/s00013-025-02105-1

**Published:** 2025-03-08

**Authors:** Scott Harper, Martin W. Liebeck

**Affiliations:** 1 School of Mathematics and Statistics, University of St Andrews, St Andrews, KY16 9SS UK; 2 Department of Mathematics, Imperial College London, London, SW7 2BZ UK

**Keywords:** Projective representation, Extensions, Simple group, 20C25, 20E32, 20C33

## Abstract

Feit and Tits (1978) proved that a nontrivial projective representation of minimal dimension of a finite extension of a finite nonabelian simple group *G* factors through a projective representation of *G*, except for some groups of Lie type in characteristic 2; the exact exceptions for *G* were determined by Kleidman and Liebeck (1989). We generalise this result in two ways. First we consider all low-dimensional projective representations, not just those of minimal dimension. Second we consider all characteristically simple groups, not just simple groups.

## Introduction

Throughout this paper, by an *extension of a group G (by a group N)* we mean a surjective group homomorphism $\gamma :H \rightarrow G$ (with kernel *N*). An extension $\gamma :H \rightarrow G$ is said to be *proper* if $\gamma$ is not an isomorphism and *minimal* if $K\gamma < G$ for all $K < H$.

Let *k* be an algebraically closed field. For a finite group *G*, let $R_k(G)$ be the minimal dimension of a nontrivial projective representation of *G* over *k*, that is, a homomorphism $\lambda :G \rightarrow \textrm{PGL}_m(k)$. In [4], Feit and Tits asked whether it was possible that an extension *H* of a finite simple group *G* could have a nontrivial projective representation $H \rightarrow \textrm{PGL}_m(k)$ where $m < R_k(G)$. The following example highlights one way that this can happen.

### Example

Assume that $\text {char}\, k \ne 2$. Let $G = \textrm{Sp}_{2n}(2)$ with $n > 2$. By [4, Theorem (I)], there exists a minimal extension *H* of *G* (by an elementary abelian group of order $2^{2n}$) that embeds irreducibly in $\textrm{PGL}_{2^n}(k)$. Hence, $R_k(H) \leqslant 2^n$, but $R_k(G) > 2^n$ (see the main theorem of [10]).

In light of this example, for a finite group *G*, define

$$\begin{aligned} n_G= \min \{ n \mid G \preccurlyeq \textrm{Sp}_{2n}(2) \text { irreducible} \}. \end{aligned}$$

Here, and throughout, $X \preccurlyeq Y$ means that the group *X* is isomorphic to a subgroup of the group *Y*.

The following is one of the main theorems of [4].

**Theorem 1 **(Feit and Tits, 1978). Let *G* be a finite nonabelian simple group, and $\gamma :H \rightarrow G$ a finite minimal extension. Let $\lambda :H \rightarrow \textrm{PGL}_m(k)$ be a nontrivial projective representation of minimal dimension. Assume that $m < 2^{n_G}$ if $\textrm{char}\, k \ne 2$. Then $\ker \gamma = \ker \lambda$.

Hence, for any finite nonabelian simple group *G*, the minimum of $R_k(H)$ across all finite extensions *H* of *G* is $\min \{R_k(G), 2^{n_G}\}$ if $\textrm{char}\, k \ne 2$, and is $R_k(G)$ if $\textrm{char}\, k = 2$. In [8], Kleidman and Liebeck determine exactly when $2^{n_G} < R_k(G)$ and hence when there exists a finite extension *H* of *G* such that $R_k(H) < R_k(G)$.

Bounds on the smallest dimension of a nontrivial projective representation of a group have many applications. Moreover, often it is useful to know about all low-dimensional representations and not just the ones of minimal dimension. There are many such results for simple groups. Denote by $\textrm{Lie}(p)$ the set of simple groups of Lie type defined over fields of characteristic *p*. Some results on low-dimensional representations of groups in $\textrm{Lie}(p)$ in $p'$-characteristic are summarised in [13], while for representations of $\textrm{Lie}(p)$ in characteristic *p*, see [12], and for alternating groups, see [6].

In view of this, it is natural to ask about the low-dimensional projective representations of extensions of simple groups. This is what our first main theorem concerns. In the statement, as usual, a projective representation $\lambda :H \rightarrow \textrm{PGL}_m(k) \cong \textrm{PGL}(V)$ is *imprimitive* if the preimage of *H* in $\textrm{GL}(V)$ permutes among themselves the subspaces $V_1$, ..., $V_m$ in a direct sum decomposition $V = \bigoplus _{i=1}^m V_i$ where $m>1$; otherwise $\lambda$ is *primitive*.

### Theorem

Let *G* be a finite nonabelian simple group, and let $\gamma :H \rightarrow G$ be a finite minimal extension. Let $\lambda :H \rightarrow \textrm{PGL}_m(k)$ be a primitive projective representation. Assume that $m < 2^{n_G}$ if $\textrm{char}\, k \ne 2$. Then $\ker \gamma \leqslant \ker \lambda$.

To state a consequence of Theorem 1, for a finite group *G*, define

$$\begin{aligned} P(G)= \min \{ d \mid G \preccurlyeq S_d \}. \end{aligned}$$

### Corollary 2

Let *G* and *H* be as in Theorem 1, and let $\lambda :H \rightarrow \textrm{PGL}_m(k)$ be an irreducible projective representation. Assume that $m<P(G)$, and if $\textrm{char}\, k \ne 2$, that $m < 2^{n_G}$. Then $\ker \gamma \leqslant \ker \lambda$.

Corollary 2 follows immediately from Theorem 1 since any irreducible representation of *H* of dimension $m<P(G)$ must be primitive. The values of *P*(*G*) for $G = G(q) \in \textrm{Lie}(p)$ are given by [9, Thm. 5.2.2] if *G* is a classical group, and by [11] if *G* is an exceptional group. In all cases, *P*(*G*) is a polynomial in *q* of degree at least the Lie rank of *G* (and in most cases much larger degree).

Corollary 2 is not true without the hypothesis $m<P(G)$, as the next example illustrates.

### Example

Let $G = A_n$ with $n > 9$ and let *p* be an odd prime dividing *n*. By [5, Theorem 1.2(i)], there exists a minimal extension *H* of *G* by an elementary abelian *p*-group *M* that embeds in $S_{\frac{1}{2}pn(n-1)}$ and hence in $\textrm{PGL}_m(k)$ for some $m < \frac{1}{2}pn(n-1)$. (The extension is minimal since, as noted in the proof of [5, Theorem 1.2(i)], *M* is contained in the Frattini subgroup of *G*.) However, $n_G \geqslant n-2$, so if $p=3$, then $m< \tfrac{3}{2}n(n-1) < 2^{n_G}$.

The next result determines the low-dimensional projective representations of extensions of simple groups of Lie type in defining characteristic.

### Corollary 3

Assume that $\textrm{char}\, k = p > 0$. Let $G \in \textrm{Lie}(p)$ and assume that $(G,p) \ne (\textrm{PSp}_4(3),3)$. Let $\gamma :H \rightarrow G$ be a finite minimal extension, and $\lambda :H \rightarrow \textrm{PGL}_m(k)$ an irreducible projective representation. If $m<P(G)$, then $\ker \gamma \leqslant \ker \lambda$.

Corollary 3 is proved in Section 3. The excluded case $(G,p) = (\textrm{PSp}_4(3),3)$ is a genuine exception as there is a minimal extension of the form $2^{6}.G \cong 2^6.\Omega ^-_6(2)$ that has a faithful irreducible projective representation of dimension 8 in characteristic 3, whereas $P(G) = 27$.

As mentioned above, if $G = G(q) \in \textrm{Lie}(p)$, then *P*(*G*) is a polynomial in *q* of degree at least the Lie rank of *G*, whereas *G* has irreducible representations of much smaller dimension than this, so Corollary 3 is quite effective in many cases. We illustrate with some examples.

### Example

(i) Let $G = \textrm{PSL}_d(q)$, excluding $\textrm{PSL}_4(2) \cong A_8$, and let $\gamma :H \rightarrow G$ be a finite minimal extension. Suppose $\lambda :H \rightarrow \textrm{PGL}_m(k)$ is an irreducible projective representation of dimension $m \leqslant \frac{1}{2}d(d+1)$, where $\textrm{char}\,k=p$ and $q=p^f$. If $d = 2$, then $P(G) > 3 = \frac{1}{2}d(d+1)$, and if $d \geqslant 3$, then $P(G) = \frac{q^d-1}{q-1} > \frac{1}{2}d(d+1)$. Hence $m<P(G)$, so Corollary 3 implies that $\ker \gamma \leqslant \ker \lambda$, which means that $\lambda$ is the lift of a projective representation of *G*. The irreducible projective representations of *G* of dimension at most $\frac{1}{2}d(d+1)$ are given in [9, Proposition 5.4.11]: $\lambda$ (or its dual) is the lift of the natural representation of *G* of dimension *d*, its alternating or symmetric square of dimension $\frac{1}{2}d(d\pm 1)$, or its alternating cube of dimension 20 if $d=6$.
(ii) If $G = E_8(q)$, then *P*(*G*) is the index of the largest parabolic subgroup, which is a polynomial in *q* of degree 57. Hence if *H* is a finite minimal extension of *G*, then every irreducible projective representation over *k* of dimension at most 100,000 is the lift of one of the representations of *G* given in [12, Table A.53].

In our next result, we extend considerations to extensions of characteristically simple groups, not just simple groups. To state it, we need to define a variant of $n_G$:

$$\begin{aligned} n'_G= \min \{ n \mid G \preccurlyeq \textrm{GL}_{2n}(2) \text { irreducible} \}. \end{aligned}$$

It is clear that $n'_G \leqslant n_G$, but, as demonstrated in Lemma 3.1, $n'_G$ is easier to work with.

### Theorem 1

Let $G = T^\ell$ for a finite nonabelian simple group *T* and a positive integer $\ell$. Let $\gamma :H \rightarrow G$ be a finite minimal extension. Let $\lambda :H \rightarrow \textrm{PGL}_m(k)$ be a faithful primitive projective representation. Assume that $m < \ell \cdot 2^{n'_T}$ if $\textrm{char}\, k \ne 2$. Then $\gamma$ is an isomorphism.

The following corollary is immediate from the theorem.

### Corollary 5

Let $G = T^\ell$ for a finite nonabelian simple group *T* and a positive integer $\ell$. Let $\gamma :H \rightarrow G$ be a finite proper minimal extension.

(i) If $\textrm{char}\, k = 2$, then *H* has no faithful irreducible primitive projective representations.
(ii) If $\textrm{char}\, k \ne 2$, then every faithful primitive projective representation of *H* has dimension at least $\ell \cdot 2^{n'_T}$.

We expect these results to have applications. Indeed, Theorem 4 has already been applied in a recent paper of Ellis and Harper [3].

## Preliminaries

### Symplectic-type *r*-groups

We use this first preliminary section to collect together some key results from [4, Section 2].

Let $V = \mathbb {F}_r^d$ where *r* is prime and let *f* be an alternating form on *V*. If $r=2$, then let *Q* be a quadratic form on *V* with bilinear form *f*. Write $\dim (V/\textrm{rad}\, V) = 2n$. As noted in [4, (2.1)], up to isomorphism, there exists a unique central extension of groups

$$\begin{aligned} 0 \rightarrow \mathbb {F}_r \rightarrow R \xrightarrow {\pi } V \rightarrow 0 \end{aligned}$$

such that for all $x, y \in R$, $[x,y] = f(x\pi ,y\pi )$ and $x^r = 1$ if $r \ne 2$ and $x^r = Q(x\pi )$ if $r=2$. Now assume that *Z*(*R*) is cyclic, which means that

1. if $r \ne 2$, then *f* is nondegenerate and the isometry group of *f* is $X = \textrm{Sp}_{2n}(r)$,
2. if $r = 2$, then *Q* is nondegenerate and the isometry group *X* of *Q* is as follows:
  - *f* is nondegenerate and $X = \textrm{O}^\epsilon _{2n}(2)$ where $\epsilon \in \{+,-\}$ is the sign of *Q*,
  - *f* has defect 1 and $X = \textrm{O}_{2n+1}(2) \cong \textrm{Sp}_{2n}(2)$.

According to these cases, we will denote *R* by

2.1 $$\begin{aligned} \text {(1)}\quad r^{1+2n} \qquad \text {(2a)}\quad 2^{1+2n}_\pm \qquad \text {(2b)}\quad 4 \circ 2^{1+2n}. \end{aligned}$$

These groups have a straightforward characterisation [4, Lemma 2.6].

#### Lemma 2.1

Let *r* be prime and let *R* be a nonabelian *r*-group all of whose proper characteristic subgroups are cyclic and central. Then *R* is isomorphic to a group in (2.1) for some *n*.

Let *R* be a group in (2.1). Write $I = \textrm{Inn}(R)$ and $A = C_{\textrm{Aut}(R)}(Z(R))$. Continue to write *X* for the associated isometry group. The following is recorded in [4, (2.2) & (2.3)].

#### Lemma 2.2

There is a short exact sequence $1 \rightarrow I \rightarrow A \rightarrow X \rightarrow 1$, which splits if $r \ne 2$.

The following is [4, (2.4) & Proposition 2.5].

#### Lemma 2.3

Let *k* be an algebraically closed field with $\textrm{char}\, k \ne r$. Let $\tau :R \rightarrow \textrm{GL}_m(k)$ be a faithful irreducible representation. Then $m=r^n$ and $N_{\textrm{GL}_m(k)}(R\tau )$ is an extension of *A* by $Z(\textrm{GL}_m(k))$.

The following is extracted from [4, (3.4)].

#### Proposition 2.4

Let *k* be an algebraically closed field, let $m \geqslant 2$ be an integer, let $H \leqslant \textrm{GL}_m(k)$ and let *N* be a normal subgroup of *H* such that

(i) *N* is nilpotent,
(ii) *N* contains the subgroup of $Z(\textrm{GL}_m(k))$ of order 4 if $\textrm{char}\, k \ne 2$,
(iii) *N* is an irreducible subgroup of $\textrm{GL}_m(k)$,
(iv) for all $M \leqslant N$ such that , either *M* is an irreducible subgroup of $\textrm{GL}_m(k)$ or *M* is a cyclic subgroup of *Z*(*H*).

Then there exists a prime $r \ne \textrm{char}\, k$ and a positive integer *n* such that $m=r^n$ and there exists a faithful irreducible representation $H/N \rightarrow \textrm{Sp}_{2n}(r)$.

#### Proof

Let $E_0$ be a minimal noncentral normal subgroup of *H* contained in *N*. Since *N* is nilpotent, $E_0$ is a *r*-group for some prime *r*. If $\textrm{char}\, k = p > 0$, then *N* has no normal *p*-subgroup since *H* is irreducible, but *N* is nilpotent, which means that *N* is a $p'$-group, so $r \ne p$. If $r \ne 2$, then let $E = E_0$, and if $r = 2$, then $E = E_0Z_4$, where $Z_4$ is the subgroup of $Z(\textrm{GL}_m(k))$ of order 4. Since *E* is noncentral, the hypotheses in the statement ensure that *E* is irreducible and hence *E* is nonabelian. Note that every proper characteristic subgroup of *E* is cyclic and central. Therefore, by Lemma 2.1, *E* is isomorphic to a group in (2.1) for some *n*. In fact, the choice of *E* ensures that $E = r^{1+2n}$ if $r \ne 2$ and $E = 4 \circ 2^{1+2n}$ if $r = 2$. Since *E* is irreducible, Lemma 2.3 implies that $m = r^n$.

Let $Y = C_{N}(E)$. Since *E* is irreducible, by Schur’s lemma, $Y \leqslant Z(\textrm{GL}_m(k))$. In particular, $Y = Z(N)$. Let $I = \textrm{Inn}(E)$ and $A = C_{\textrm{Aut}(E)}(Z(E))$. Let ${\widetilde{A}} = N_{\textrm{GL}_m(k)}(E)$, noting that, by Lemma 2.3, we have ${\widetilde{A}}/Z(\textrm{GL}_m(k)) = A$. In particular, we have

$$\begin{aligned} EY \leqslant N \leqslant H \leqslant {\widetilde{A}} \leqslant \textrm{GL}_m(k) \end{aligned}$$

and $I = EY/Y \leqslant A \leqslant \textrm{PGL}_m(k)$. Since ${\widetilde{A}}/EY \cong A/I \cong \textrm{Sp}_{2n}(r)$, we obtain a faithful representation $\mu :H/EY \rightarrow \textrm{Sp}_{2n}(r)$.

We claim that $(H/EY)\mu$ is irreducible. To see this, let *U* be a proper subgroup of *EY*/*Y* that is normalised by *H*. Noting that $EY/Y \cong E_0/(E_0 \cap Y)$, let $U_0$ be the corresponding subgroup of $E_0$, which is normalised by *H*. Since $E_0$ was chosen to be a minimal noncentral normal subgroup of *H* contained in *N*, $U_0$ is central in *H*. In particular, *U* is trivial.

We claim that $N = EY$. Since *N* is nilpotent, *N*/*Y* is an *r*-group, so *N*/*EY* is an *r*-group too. However, since $(H/EY)\mu$ is irreducible, *H*/*EY* has no nontrivial normal *r*-subgroup, so $N = EY$. Therefore, $\mu :H/N \rightarrow \textrm{Sp}_{2n}(r)$ is a faithful irreducible representation, as required. $\square$

### Subdirect products

We conclude this preliminary section with a technical lemma regarding subdirect products.

#### Lemma 2.5

Let *H* be a subdirect product of $H_1 \times H_2$ with projections $\pi _1:H \rightarrow H_1$ and $\pi _2:H \rightarrow H_2$. Assume that *N* is a soluble normal subgroup of *H* such that $H/N \cong T^\ell$ where *T* is a nonabelian simple group and $\ell$ is a positive integer. Then there exist integers $\ell _1, \ell _2 \geqslant 0$ satisfying $\ell _1 + \ell _2 \geqslant \ell$ such that $H_1/N\pi _1 \cong T^{\ell _1}$ and $H_2/N\pi _2 \cong T^{\ell _2}$.

#### Proof

Let $i \in \{1,2\}$. Write $K_i = \ker \pi _i$. Since , write $NK_i/N \cong T^{m_i}$ for some $m_i \leqslant \ell$. Now

$$\begin{aligned} H_i/N\pi _i \cong (H/K_i)/(NK_i/K_i) \cong H/NK_i \cong (H/N)/(NK_i/N) \cong T^{\ell -m_i} \end{aligned}$$

since $H/N \cong T^\ell$ and $NK_i/N \cong T^{m_i}$. Write $\ell _i = \ell -m_i$. We claim that $m_1+m_2 \leqslant \ell$, which implies that $\ell _1+\ell _2 \geqslant \ell$, as required.

To prove the claim, first note that

$$\begin{aligned} K_i/(K_i \cap N) \cong NK_i/N \cong T^{m_i} \end{aligned}$$

and $K_i \cap N$ is soluble, so $K_i \cap N$ is the soluble radical of $K_i$. Since $K_1 \cap K_2 = 1$, we have $K_1K_2 \cong K_1 \times K_2$, which implies that $(K_1 \cap N)(K_2 \cap N)$ is the soluble radical of $K_1K_2$. Since

$$\begin{aligned} K_1K_2/(K_1K_2 \cap N) \cong K_1K_2N/N \leqslant H/N \cong T^\ell , \end{aligned}$$

write $K_1K_2/(K_1K_2 \cap N) \cong T^m$ for some $m \leqslant \ell$. Since $K_1K_2 \cap N$ is soluble, $K_1K_2 \cap N$ is the soluble radical of $K_1K_2$, so $K_1K_2 \cap N = (K_1 \cap N)(K_2 \cap N)$. Therefore,

$$\begin{aligned} T^m \cong K_1K_2/(K_1K_2 \cap N) \cong K_1/(K_1 \cap N) \times K_2/(K_2 \cap N) \cong T^{m_1} \times T^{m_2}, \end{aligned}$$

so $m_1 + m_2 = m \leqslant \ell$, as claimed. $\square$

#### Corollary 2.6

Let *H* be a subdirect product of $H_1 \times \cdots \times H_r$ and let $\pi _i:H \rightarrow H_i$ be the projection onto the *i*th factor. Assume that *N* is a soluble normal subgroup of *H* such that $H/N \cong T^\ell$ where *T* is a nonabelian simple group and $\ell$ is a positive integer. Then there exist nonnegative integers $\ell _1, \dots , \ell _r$ satisfying $\ell _1 + \cdots + \ell _r \geqslant \ell$ such that $H_i/N\pi _i \cong T^{\ell _i}$ for all $1 \leqslant i \leqslant r$.

#### Proof

We proceed by induction on *r*, noting that the result certainly holds when $r=1$. Now assume that $r > 1$. Let $\pi _0:H \rightarrow H_2 \times \cdots \times H_r$ be the projection to $H_2 \times \cdots \times H_r$ and let $H_0 = \textrm{im}\pi _0$. Then *H* is a subdirect product of $H_1 \times H_0$. Therefore, by Lemma 2.5, we can fix integers $\ell _1, \ell _0 \geqslant 0$ satisfying $\ell _1+\ell _0 \geqslant \ell$ such that $H_1/N\pi _1 = T^{\ell _1}$ and $H_0/N\pi _0 = T^{\ell _0}$. Since $N\pi _0$ is soluble and $H_0$ is a subdirect product of $H_2 \times \cdots \times H_r$, by induction, there exist integers $\ell _2, \dots , \ell _r \geqslant 0$ satisfying $\ell _2 + \cdots + \ell _r \geqslant \ell _0$ such that $H_i/N\pi _i = H_i/N\pi _0\pi _i \cong T^{\ell _i}$ for all $2 \leqslant i \leqslant r$. Therefore, $\ell _1 + \cdots + \ell _r \geqslant \ell$ and $H_i/N\pi _i \cong T^{\ell _i}$ for all $1 \leqslant i \leqslant r$, which completes the induction. $\square$

## Proofs

We now prove our main results. The proofs of Theorems 1 and 4 are both inspired by the proof of [4, Theorem (II’)].

### Proof of Theorem 1

For a contradiction, suppose otherwise, and choose *m* minimally for a counterexample. Let $N = \ker \gamma$.

We claim that *N* is the unique maximal normal subgroup of *H*. To see this, let *K* be a proper normal subgroup of *H*. Since $K < H$, we have $K\gamma < G$, so $K\gamma = 1$ since *G* is simple. Therefore, $K \leqslant N$, as claimed.

In particular, $\ker \lambda \leqslant N$, so *G* is a quotient of $H\lambda$. Therefore, by replacing *H* by $H\lambda$, we may assume that $\lambda$ is faithful. Let $\eta :{\widetilde{H}} \rightarrow H$ be a finite central extension such that $\lambda$ lifts to a faithful representation ${\widetilde{\lambda }}:{\widetilde{H}} \rightarrow \textrm{GL}_m(k)$, and, if $\text {char}\, k \ne 2$, then choose ${\widetilde{H}}$ such that ${\widetilde{H}}{\widetilde{\lambda }}$ contains the subgroup $Z_4$ of $Z(\textrm{GL}_m(k))$ of order 4. Let ${\widetilde{\gamma }} = \eta \gamma$ and let ${\widetilde{N}} = \ker {\widetilde{\gamma }}$.

We claim that ${\widetilde{N}}$ is nilpotent. Let *S* be a Sylow subgroup of *N*. By the Frattini argument, $H = N_H(S)N$, so $N_H(S)\gamma = G$, which implies that $H = N_H(S)$, so . Therefore, *N* is nilpotent, and ${\widetilde{N}}$, being a central extension of *N*, is also nilpotent.

Let ${\widetilde{M}} \leqslant {\widetilde{N}}$ be such that . We claim that either ${\widetilde{\lambda }}_{{\widetilde{M}}}$ is irreducible or ${\widetilde{M}}$ is a cyclic subgroup of $Z(\textrm{GL}_m(k))$. Since ${\widetilde{\lambda }}$ is irreducible, by Clifford’s theorem, we can write ${\widetilde{\lambda }}_{{\widetilde{M}}} = \alpha _1 \oplus \cdots \oplus \alpha _k$ where $\alpha _1, \dots , \alpha _k$ are the homogenous components of ${\widetilde{\lambda }}_{{\widetilde{M}}}$ whose respective irreducible components are pairwise nonisomorphic. Moreover, ${\widetilde{H}}$ acts transitively on these *k* components, and so, since by hypothesis the representation ${\widetilde{\lambda }}$ is primitive, we have $k=1$. Thus ${\widetilde{\lambda }}|_{{\widetilde{M}}} = \alpha _1$, which is the direct sum of $m_1$ isomorphic irreducible representations of dimension $m_2$. Therefore, $\lambda = \lambda _1 \otimes \lambda _2$ where $\lambda _1:H \rightarrow \textrm{PGL}_{m_1}(k)$ and $\lambda _2:H \rightarrow \textrm{PGL}_{m_2}(k)$ are irreducible projective representations of *H* (see [2, Theorem 3]). Suppose that $1< m_1 < m$ and $1< m_2 < m$. The minimality of *m* means that $\ker \gamma \leqslant \ker \lambda _1$ and $\ker \gamma \leqslant \ker \lambda _2$, so $\ker \gamma \leqslant \ker \lambda$, which is a contradiction.

Therefore, we can assume that either $m_1=1$ or $m_2=1$, which is to say, either ${\widetilde{\lambda }}_{{\widetilde{M}}}$ is irreducible, or ${\widetilde{\lambda }}_{{\widetilde{M}}}$ a direct sum of isomorphic linear representations. In the latter case, ${\widetilde{M}}$ is represented by scalars, so ${\widetilde{M}}$ is a cyclic subgroup of $Z(\textrm{GL}_m(k))$.

We claim that ${\widetilde{\lambda }}_{{\widetilde{N}}}$ is irreducible. Suppose otherwise. Then the previous paragraph implies that ${\widetilde{N}} \leqslant Z(\textrm{GL}_m(k))$, so $\ker \gamma = N = {\widetilde{N}}\eta = 1$, which is a contradiction.

Therefore, Proposition 2.4 yields a faithful irreducible representation $\mu : G \rightarrow \textrm{Sp}_{2n}(r)$ where $m = r^n$ for a prime $r \ne \textrm{char}\, k$. By Lemma 2.2, if $r \ne 2$, then *H* is a split extension of *G*, so $\ker \gamma = 1$, which is a contradiction. Therefore, $r = 2$, so $\textrm{char}\, k \ne 2$ and $m = 2^n \geqslant 2^{n_G}$, which contradicts our assumption that $m < 2^{n_G}$. $\square$

### Proof of Corollary 3

Let $G \in \textrm{Lie}(p)$, and let $\gamma :H \rightarrow G$ be a finite minimal extension. The conclusion follows from Corollary 2 if $p=2$, so assume that $p\ne 2$.

**Table 1:** .

*G*	*P*(*G*)	$n_G$
$\textrm{PSL}_2(17)$	18	4
$\textrm{PSp}_4(3)$	27	3
$\textrm{PSU}_3(3)$	28	3
$G_2(3)$	351	7

We first claim that either $P(G) \leqslant 2^{n_G}$ or *G* is as in Table 1. It is a routine matter to verify the claim. The values of *P*(*G*) are given by [9, Thm. 5.2.2] if *G* is a classical group, and by [11] when *G* is an exceptional group of Lie type; while $n_G \geqslant \frac{1}{2}R_{p'}(G)$, lower bounds for which can be found in [9, Table 5.3.A]. Comparison of these bounds gives $P(G) \leqslant 2^{n_G}$ apart from some small simple groups *G* (including those in the table above), for which the precise values of $n_G$ can be read off using [7]. The claim follows.

Given the claim, the conclusion of Corollary 3 follows from Corollary 2, provided we rule out the groups *G* in the above table. The group $\textrm{PSp}_4(3)$ is excluded by hypothesis. Now consider $G = \textrm{PSL}_2(17)$ (resp. $\textrm{PSU}_3(3)$, $G_2(3)$). From [7], we see that the nontrivial irreducible $\mathbb {F}_2G$-modules of dimension at most $2\log _2(P(G))$ have dimensions 8 (resp. 6, 14). Suppose $\lambda :H \rightarrow \textrm{PGL}_m(k)$ is irreducible of dimension $m<P(G)$ with $\ker \gamma \not \leqslant \ker \lambda$. Then it follows from the proof of Theorem 1 that $K:=\ker \gamma = 2^8$ (resp. $2^6$, $2^{14}$) and $m=16$ (resp. 8, 128). Now *H* must be a nonsplit extension of *G* by *K*. However, a computation in Magma [1] shows that $H^2(G,K)=0$ for $G = \textrm{PSL}_2(17)$, $G_2(3)$, which rules out these groups. The last observation does not apply to $G = \textrm{PSU}_3(3)$ (as $H^2(\textrm{PSU}_3(3),2^6)) \ne 0$); however, for this case we have $H\lambda = 2^6.\textrm{PSU}_3(3)< \textrm{PGL}_8(9) < \textrm{PGL}_8(k)$, whereas a Magma computation reveals that this extension $2^6.\textrm{PSU}_3(3)$ splits. This completes the proof. $\square$

Before proving Theorem 4, we need a result that highlights a key property of the invariant $n_G'$ defined in the introduction.

### Lemma 3.1

Let $G = T^\ell$ for a nonabelian finite simple group *T* and $\ell \geqslant 1$. Then $n_G' \geqslant n_T' \cdot 2^{\ell -1}$.

### Proof

The definition of $n_G'$ guarantees the existence of a faithful irreducible representation $\rho :G \rightarrow \textrm{GL}_{2n_G'}(2)$. Let *V* be the $\mathbb {F}_2G$-module afforded by $\rho$, let $E = \textrm{End}_{\mathbb {F}_2G}(V)$, and let $e = |E:\mathbb {F}_2|$. Then *e* divides $2n_G'$ and there exists a faithful absolutely irreducible representation $\rho _1 :G \rightarrow \textrm{GL}_{2n'_G/e}(2^e)$ (see [9, Lemma 2.10.2]). Since $G = T^\ell$, there exists a faithful absolutely irreducible representation $\rho _2 :T \rightarrow \textrm{GL}_n(2^e)$ where $n^\ell = 2n_G'/e$ (see [9, Lemma 5.5.5]). Let *f* be minimal such that $\rho _2$ is expressible over the subfield $\mathbb {F}_{2^f} \subseteq \mathbb {F}_{2^e}$, and consider the corresponding faithful absolutely irreducible representation $\rho _3 :T \rightarrow \textrm{GL}_n(2^f)$. Via the field extension embedding $\textrm{GL}_n(2^f) \preccurlyeq \textrm{GL}_{fn}(2)$, we obtain a faithful irreducible representation $\rho _4:T \rightarrow \textrm{GL}_{fn}(2)$. Therefore, $fn \geqslant 2n_T'$. We now conclude that

$$\begin{aligned} 2n_T' \leqslant fn \leqslant en \leqslant \frac{en^\ell }{2^{\ell -1}} \leqslant \frac{n_G'}{2^{\ell -2}}, \end{aligned}$$

and hence $n_G' \geqslant n_T' \cdot 2^{\ell -1}$, as required. $\square$

### Proof of Theorem 4

For a contradiction, suppose otherwise, and choose *m* minimally for a counterexample. Let $N = \ker \gamma$.

Since $\lambda$ is faithful, by replacing *H* with $H\lambda$, we assume that $H \leqslant \textrm{PGL}_m(k)$. Let $\eta :{\widetilde{H}} \rightarrow H$ be a finite central extension such that ${\widetilde{H}} \leqslant \textrm{GL}_m(k)$, and, if $\textrm{char}\, k \ne 2$, then choose ${\widetilde{H}}$ to contain the subgroup $Z_4$ of $Z(\textrm{GL}_m(k))$ of order 4. Let ${\widetilde{\gamma }} = \eta \gamma$ and let ${\widetilde{N}} = \ker {\widetilde{\gamma }}$.

As in the proof of Theorem 1, ${\widetilde{N}}$ is nilpotent. Let ${\widetilde{M}} \leqslant {\widetilde{N}}$ be such that . We claim that either ${\widetilde{M}}$ is irreducible or ${\widetilde{M}}$ is a cyclic subgroup of $Z({\widetilde{H}})$. Since ${\widetilde{H}}$ is primitive, by Clifford’s theorem, the representation of ${\widetilde{M}}$ is the direct sum of $m_1$ isomorphic irreducible representations of dimension $m_2$. Therefore, $\lambda = \lambda _1 \otimes \lambda _2$ where $\lambda _1:H \rightarrow \textrm{PGL}_{m_1}(k)$ and $\lambda _2:H \rightarrow \textrm{PGL}_{m_2}(k)$ are irreducible projective representations of *H* (see [2, Theorem 3]). Moreover, $\lambda _1$ and $\lambda _2$ are both primitive since $\lambda$ is.

Suppose that $1< m_1 < m$ and $1< m_2 < m$. For $i \in \{1,2\}$, let $K_i = \ker \lambda _i$ and $H_i = H/K_i$, and note that we have a faithful primitive projective representation of $H_i$ afforded by the embedding $H_i \cong \textrm{im}\lambda _i \leqslant \textrm{PGL}_{m_i}(k)$. Let $N_i = NK_i/K_i$ and let $\gamma _i:H_i \rightarrow H_i/N_i$ be the corresponding quotient map. Now *H* is isomorphic to a subdirect product of $H_1 \times H_2$, so Lemma 2.5 implies that $H_i/N_i = T^{\ell _i}$ for integers $\ell _1,\ell _2 \geqslant 0$ such that $\ell _1 + \ell _2 \geqslant \ell$. Suppose that there exists $i \in \{1,2\}$ and $X < H_i$ such that $X\gamma _i = H_i/N_i$. Then $XN_i/N_i = H_i/N_i$, so $XN_i = H_i$. Since $H_i = H/K_i$, we can write $X = Y/K_i$ for some $K_i \leqslant Y < H$. Then $(Y/K_i) (NK_i/K_i) = H/K_i$, so $YN = H$ since $K_i \leqslant Y$. By assumption, this means that $Y = H$, so $X = H_i$, which is a contradiction. Therefore, for $i \in \{1,2\}$, if $X < H_i$, then $X\gamma _i < H_i/N_i$. For now assume that $\textrm{char}\, k \ne 2$. We claim that $m_i < \ell _i \cdot 2^{n_T'}$ for $i \in \{1,2\}$. Write $\{i,j\} = \{1,2\}$. Suppose that $m_i \geqslant \ell _i \cdot 2^{n_T'}$. Since $m < \ell \cdot 2^{n_T'}$, we have

$$\begin{aligned} m_j = m/m_i < \ell /\ell _i \leqslant 2(\ell -\ell _i) \leqslant 2\ell _j. \end{aligned}$$

Note that $m_j < \ell _j \cdot 2^{n_T'}$ since $n_T' \geqslant 1$. Therefore, the minimality of *m* implies that $\gamma _j$ is an isomorphism, so $T^{\ell _j} = H_j$ is an irreducible subgroup of $\textrm{PGL}_{m_j}(k)$. Writing $d_p(T)$ for the smallest dimension *d* of an irreducible projective representation $T \rightarrow \textrm{PGL}_d(k)$, by [9, Proposition 5.5.7(ii)], we have $m_j \geqslant d_p(T)^{\ell _j} \geqslant 2^{\ell _j} \geqslant 2\ell _j$, which contradicts the displayed inequality. Therefore, $m_i < \ell _i \cdot 2^{n_T'}$ as claimed. Since we have verified all of the conditions of the statement, the minimality of *m* implies that $\gamma _1$ and $\gamma _2$ are isomorphisms. This implies that $N \leqslant K_1$ and $N \leqslant K_2$, so $N \leqslant K_1 \cap K_2 = 1$, which is a contradiction.

Therefore, we can assume that either $m_1=1$ or $m_2=1$, which is to say, either ${\widetilde{M}}$ is irreducible, or ${\widetilde{M}}$ is a direct sum of isomorphic linear representations. In the latter case, ${\widetilde{M}}$ is represented by scalars, so ${\widetilde{M}}$ is a cyclic subgroup of $Z(\textrm{GL}_m(k))$.

We claim that ${\widetilde{\lambda }}_{{\widetilde{N}}}$ is irreducible. Suppose otherwise. Then the previous paragraph implies that ${\widetilde{N}} \leqslant Z(\textrm{GL}_m(k))$, so $\ker \gamma = N = {\widetilde{N}}\eta = 1$, which is a contradiction.

Therefore, Proposition 2.4 yields a faithful irreducible representation $\mu : G \rightarrow \textrm{Sp}_{2n}(r)$ where $m = r^n$ for a prime $r \ne \textrm{char}\, k$. By Lemma 2.2, if $r \ne 2$, then *H* is a split extension of *G*, so $\ker \gamma = 1$, which is a contradiction. Therefore, $r = 2$, so $\textrm{char}\, k \ne 2$ and, using Lemma 3.1,

$$\begin{aligned} m = 2^n \geqslant 2^{n_G} \geqslant 2^{n_G'} = 2^{2^{\ell -1}(n_T')^\ell } \geqslant \ell \cdot 2^{n_T'}, \end{aligned}$$

which contradicts our assumption that $m < \ell \cdot 2^{n_T'}$. $\square$
